# Gold-Hybridized Zinc Oxide Nanorods as Real-Time Low-Cost NanoBiosensors for Detection of virulent DNA signature of HPV-16 in Cervical Carcinoma

**DOI:** 10.1038/s41598-019-53476-9

**Published:** 2019-11-19

**Authors:** Thevendran Ramesh, Kai Loong Foo, Haarindraprasad R., Annie Jeyachristy Sam, Maheswaran Solayappan

**Affiliations:** 1 Department of Biotechnology, Faculty of Applied Sciences, AIMST University, 08100 Semeling, Kedah Malaysia; 2 Faculty of Engineering and Computer Technology, AIMST University, 08100 Semeling, Kedah Malaysia; 3 Department of Biochemistry, Faculty of Medicine, AIMST University, 08100 Semeling, Kedah Malaysia; 4 Nano Biochip Research Group, Institute of Nano Electronic Engineering (INEE), Universiti Malaysia Perlis (UniMAP), Kangar, Perlis 01000 Malaysia

**Keywords:** Tumour virus infections, Oncogenes

## Abstract

Detection of host integrated viral oncogenes are critical for early and point-of-care molecular diagnostics of virus-induced carcinoma. However, available diagnostic approaches are incapable of combining both cost-efficient medical diagnosis and high analytical performances. To circumvent this, we have developed an improved IDE-based nanobiosensor for biorecognition of HPV-16 infected cervical cancer cells through electrochemical impedance spectroscopy. The system is fabricated by coating gold (Au) doped zinc oxide (ZnO) nanorods interfaced with HPV-16 viral DNA bioreceptors on top of the Interdigitated Electrode (IDE) chips surface. Due to the concurrently improved sensitivity and biocompatibility of the designed nanohybrid film, Au decorated ZnO-Nanorod biosensors demonstrate exceptional detection of HPV-16 E6 oncogene, the cancer biomarker for HPV infected cervical cancers. This sensor displayed high levels of sensitivity by detecting as low as 1fM of viral E6 gene target. The sensor also exhibited a stable functional life span of more than 5 weeks, good reproducibility and high discriminatory properties against HPV-16. Sensor current responses are obtained from cultured cervical cancer cells which are close to clinical cancer samples. Hence, the developed sensor is an adaptable tool with high potential for clinical diagnosis especially useful for economically challenged countries/regions.

## Introduction

**Figure 1:**
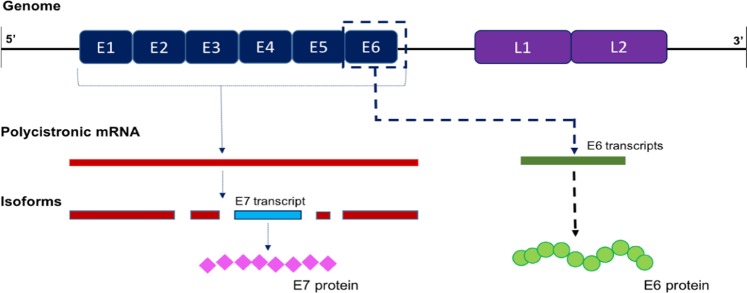
Mechanisms of E6 and E7 oncogene synthesis through transcription of polycistronic mRNA and spliced transcript isoforms.

Human Papillomavirus (HPV), is the prime causative agent in the development of cervical cancers predominantly occurring within underdeveloped, third world countries. Such cancer-inducing viral factors account for more than 57% mortality rates among the female population compared to other female relevant diseases such as breast cancer, ovarian cancer or even bacterial or fungal genital tract infections^1,2^. Currently, more than 200 HPV subtypes have been identified, among which 14 subtypes being associated with high-risk lower genital tract infections. HPV subtype-16 (HPV-16) is the most prominent amidst these infective strands which result in uncontrolled proliferation of cervical tumors and subsequent epithelial malignancy^1,3^. HPV-16 possesses specific tumor-promoting oncogenes, referred to as E6 oncogenes that facilitate the initiation of cervical carcinoma through the incorporation of viral genes within the host’s genomic DNA^4,5^. Expropriation of the host cell’s protein synthesis machinery through such viral integrations results in the expression of E6 oncoproteins (shown in Fig. 1) that lead to silencing of major tumor-suppressing proteins in humans such as p53, pRb, and p21^1,2,5,6^. Deactivation of these two proteins further disrupts important checkpoints in the cell cycle’s control mechanisms, leading to uncontrolled mitosis and the systemic progression of different stages of cervical cancer^1,2,5,7^.

Accessible diagnostic techniques such as Papanicolaou test (Pap-Smear), Hybrid Capture 2 (HC2) assays, dot blot assays, and certain nucleic acid amplification methods assist in early detections of cervical cancerous growths. However, such techniques are short on several critical aspects such as lack of specificity against HPV-16 specific factors, inability to identify dormant viral oncogenes, providing inadequate information that is skewed towards qualitative analysis and consuming more time in producing accurate and reliable data^8–10^. Diagnostic systems based on biosensor designs, tailored to detect a wide variety of molecular analytes give us better leverage over these other conventional detection methods. Hence, in this work, we present the development of a real-time, low-cost electrochemical biosensor system for rapid and accurate detection of HPV-16 viral DNA strands within infect cervical cancer samples. Here, we have described the use of interdigitated electrode (IDE) sensor platforms fabricated with an aptitude for sensitive and specific detection of HPV-16 infected cervical cells. IDE-based biosensors were chosen due to their device miniaturization offering multiple benefits such as portability, ease of modification and greater result reproducibility^11^.

Our biosensor system utilizes short, thiolated-probe sequences complementary to the E6 oncogene as the biorecognition element or capture probe of HPV-16 viruses since it is known that HPV-16 closely associates with cervical epithelial tissue through genomic integration of E6 virulent genes. The IDE chip’s active sensing area is coated with gold nanoparticle (AuNP) doped ZnO-Nanorods which provide the main functional architecture for both rapid and quantitative detection of HPV-16 in cervical cancer samples. Growth of ZnO-Nanorods provides enhanced sensitivity for analyte detection by conferring an increased total probe surface coverage and lower limit of detection (LOD). Doping of ZnO-Nanorods with nanometallic gold particles provide not only provides improved film conductivity and better sensitivity but also bestows chemisorptive properties due to the gold-thiol group interactions leading to a strong biointerfacing between the E6 bioreceptors and the proposed IDE sensor chips. Such traits allow the nanostructured film to act as an excellent transducer layer for deciphering even minute changes in biochemical signals produced during viral analyte detection. Electrochemical impedance spectroscopy was used to measure and quantify HPV-16 E6 viral DNA strands within cervical cancer cell samples. Other auxiliary aspects of the developed platform were also assessed further such as the chips sensitivity and specificity against HPV-16 viral strands as well as its stability and reproducibility. Our biosensor system not only yields a capable, low-cost sensor device but also provides a portable tool for HPV-16 detection in regions with poor diagnostic facilities.

## Results and Discussion

### ***In-sillico*** analysis of HPV-16 E6 probe’s biocapture properties

**Figure 2:**
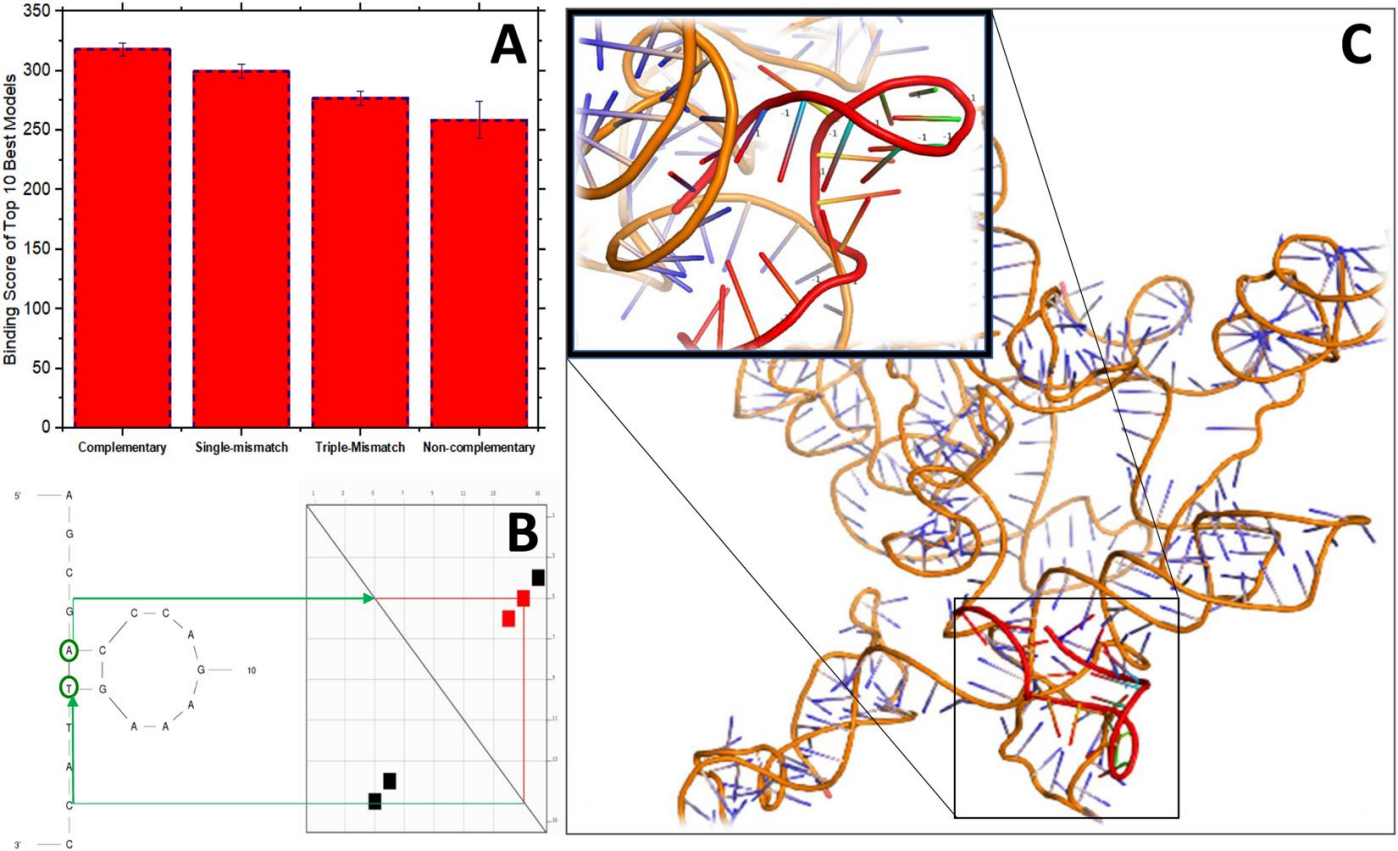
(**A**) Bar chart representing the analyzed docking score of top 10 best binding models from HNADOCK webserver of different primer sequences against target sequence. (**B**) Diagram showing the E6 capture probes MFold predicted secondary structure (left) and energy dot plot diagram (right). (**C**) Diagram of most probable binding model between E6 probes secondary conformation and targeted gDNA sequence.

*In-silico* computations were done using multiple bioinformatic platforms/tools to get a defined picture of the custom made E6 capture probe’s biophysical and target binding properties as a pre-analysis step before being synthesized and ordered from IDT.inc. MFold webserver was used foremost to predict the designed forward primer’s (E6 capture probe) secondary structure as shown in Fig. 2. This secondary structure possessed free energy, ∆G value of 2.15 Kcal/mol and was observed to have a single looped structure. Since the predicted secondary structure has a positive ∆G value (∆G > 0) attributing to a non-spontaneous structure formation, it is relatively less stable and can easily degenerate allowing the probe sequence to return to their single-stranded DNA conformation during targeted viral DNA binding, either in *in-vitro* conditions or in surface-bound/immobilized states. To additionally confirm that the predicted secondary structure is the most probable conformation that the custom-made probe can assume in a free or bound state, energy dot plot diagrams were done as seen in Fig. 2. From the plot, we can observe that only the base pairs at 5^th^ and 6^th^ positions have the most possible binding or optimal binding energy (colored as red) and form intrabase pairing. As a result, it subsequently confirms the predicted secondary structure formation and further reassures that the designed primer sequence is a suitable choice as the capture element for the proposed sensor system. Furthermore, virtual PCR runs/simulations in Genome Compiler also revealed the high specificity of the designed primer towards targeted HPV-16 E6 viral gene. As portrayed in the virtual PCR gen run, the software predicted a 134 bp amplicon being produced from PCR runs using the designed primer sequences. This specifically occurred only with HPV-16 E6 gene targets and not for HPV-18 E6 gene sequences indicating high discriminatory properties of the primers.

**Figure 3:**
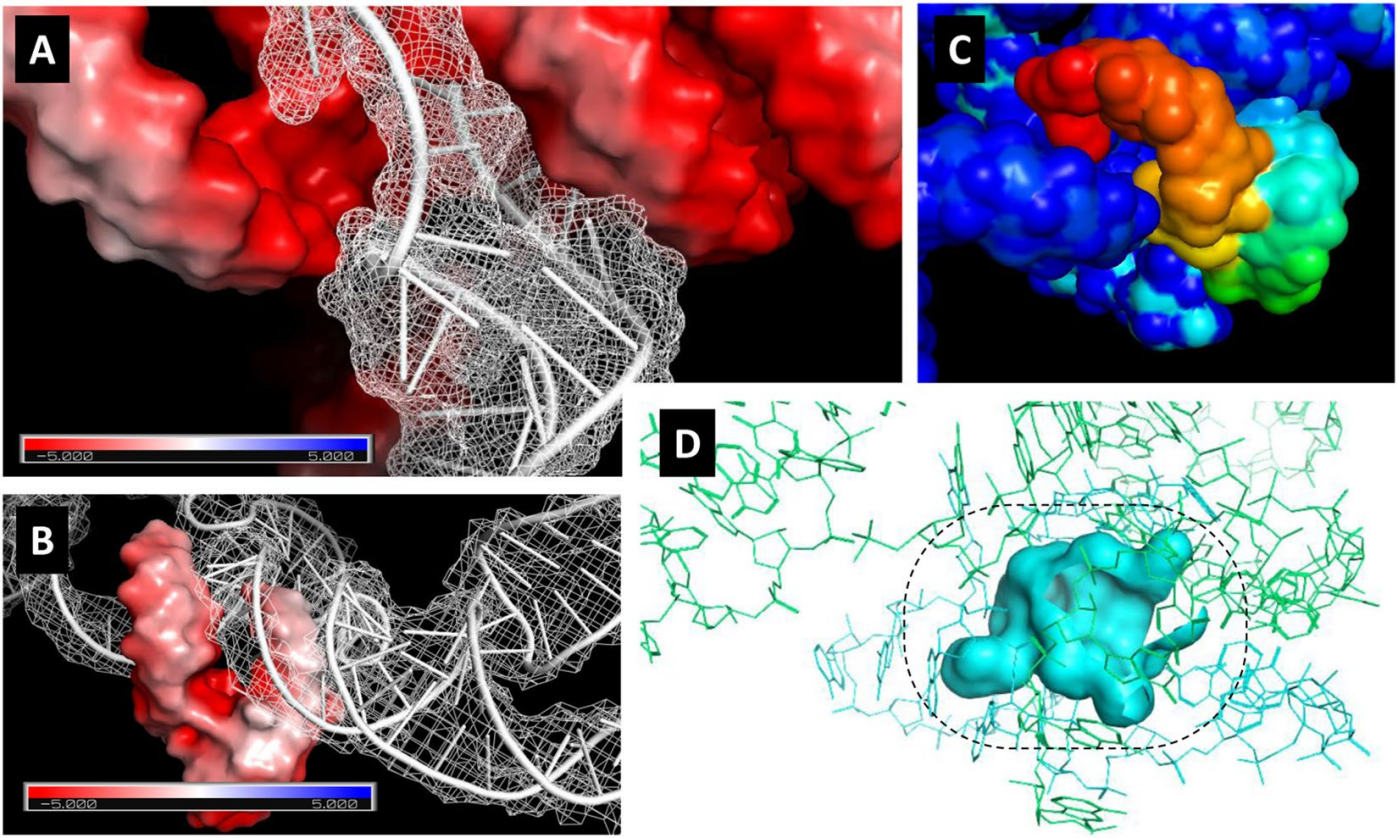
(**A**,**B**) Shows the electrostatic potential or charge distribution of 3D-gDNA and E6 probe models visualized through PyMol software. (**C**) Diagram of total solvent accessible surface area of the E6 capture probe. (**D**) Stick and Ball model visual of predicted water pocket within interacting region E6 probes secondary structure (blue) and gDNA target sequence (green).

Without a doubt, binding of the DNA-based capture probe towards the viral E6 gene is bound to occur since the design E6 probe is completely complementary to the E6 viral gene. However, one must note that in nature individual nucleic acid sequences assume different secondary structures based on free energy and physical properties bestowed by their own sequence uniqueness. Although the designed probe sequence has a relatively simple secondary structure which can easily linearize during target binding, conformations assumed by the DNA probe either in a free solution state or bound/immobilized state will, either way, influence their biocapture properties making it unavailable for E6 gene binding. Despite this, such phenomena are negligible since it is shown that sequence binding evidently still occurs when it is practically tested. We suspect that even though in-theory, secondary structure formations can deplete the number of available surface-bound capture probes, the interaction of the secondary structure units themselves with the targeted regions of gDNA provide an auxiliary means of probe-gene interactions while base complementarity serving as the main driving force. This could very well be the reason that probe binding properties are able to be retained under experimental conditions even when a small population of immobilized probe folds into their secondary conformations. Hence, HNADOCK webserver algorithms^12^ were used to analyze and observe the binding efficiency of the E6 probe’s secondary structure towards the clinical gDNA cancer samples. Through careful consideration of the top 10 best binding modes generated by the server, the probe-gene binding model shown in Fig. 3 was chosen as the most probable 3D binding conformation assumed by the two sequences during target binding. In parallel, we have also used the same *in-sillico* approach to perform control docking tests using slightly different probe sequences; single-mismatch, triple-mismatch, and non-complementary probe sequences. This was done in order to study the correlation between different nucleotide sequences and their corresponding secondary structure formation against their binding score with the target E6 viral gene.

The set of docking or binding score obtained for each different capture probe sequence from their respective top 10 best models revealed that the secondary structure generated by the complementary probe sequence possessed the highest binding score, indicative of its high target binding affinity. A predictable descending pattern of binding/docking score was seen for the other sequences, from the single-mismatch sequence, triple-mismatch and to the non-complementary sequence. The statistical significance between the docking score value of the complementary probe against the other probe sequences were analyzed using independent sample t-test analysis. The computed data showed that the difference of binding/docking score between the complementary probe sequence and the other three sequences is significantly different since the P-values obtained from all three comparisons were <0.05α shown in Table 1. This further consolidates the previous explanation that a strong interdependence exists between sequence complementarity and secondary structure formation with the capture probe’s target binding affinity. It also proves that a complementary probe sequence still holds the greatest potential for effective target binding in both its linearized and folded conformation compared to other slightly less complementary and non-complementary probe sequences.

**Table 1:** The binding score of the top 10 best models of different E6 primer secondary conformation’s having different sequences and computed *p*-values through t-test analysis.

Binding Models	Complementary Sequence	Single-Base Mismatch Sequence	Triple-Base Mismatch Sequence	Non-Complementary Sequence
Binding Score for Model 1	−344.57	−338.37	−305.9	−283.2
Binding Score for Model 1	−327.85	−311.52	−290.5	−267.7
Binding Score for Model 3	−320.98	−309.09	−285.5	−265.2
Binding Score for Model 4	−320.92	−301.28	−283.7	−259
Binding Score for Model 5	−316.84	−299.25	−279.6	−258
Binding Score for Model 6	−312.19	−295.6	−273	−257.8
Binding Score for Model 7	−306.07	−293.5	−267.7	−254.2
Binding Score for Model 8	−302.88	−288.13	−264.9	−251.3
Binding Score for Model 9	−302.8	−279.48	−262.3	−245.7
Binding Score for Model 10	−299.1	−278.26	−256.6	−243
**t-Test Computations**	**Complementary-Single mismatch**	**Complementary-Triple mismatch**	**Complementary-Non-complementary**	
P-values	0.03712	1.277 × 10^−5^	1.103 × 10^−8^	

Furthermore, the surface and physical properties of the E6 probe’s folded conformation were also analyzed in order to scrutinize their contribution to their binding properties, shown in Fig. 3. Using the binding model in the previous Fig. 2, we were able to visualize the presence of water pockets/molecules present within the probes folded conformations as well as the target viral gene region. Water molecules accumulated around the probe sequence will usually be expelled during the sequence hybridization. But the visualized water pockets show that some water molecules are not discharged and become trapped at the binding interface leading to an enthalpically favorable binding. Since any enthalpy driven biomolecular interactions are highly favorable, it clearly shows that E6 probe sequence is capable of establishing strong, specific interactions with the targeted region compared to the surrounding water molecules even in its folded conformation^13–15^. The solvent accessible surface area of the E6 probe’s folded conformation was also computed to be around ~3980 Å using a radius of 1.7683 Å^16^. This shows that most of the nucleotide residues at the hybridization interface are well exposed to the surrounding solvent environment which helps facilitate binding with targeted viral gene integrated within a large, compact human gDNA strand^17^. Fig. 3(A,B) also portrays the electrostatic potential of the probe-gene hybridized model where the red region highlights negatively charged, white being neutral, and blue being positively charged. The probes folded structure has a relatively neutral potential at its interacting surface while the targeted gene displays a strong negative charge at the binding region. As a result, electrostatic repulsion between the capture probe and the targeted viral gene can be excluded since such modes of interactions are unfavorable or nonspecific. This additionally confirms that the designed E6 probe either in its extended/folded conformation is driven mainly by base-pair or water-mediated hydrogen bonding^17,18^. Such preferred surface properties attribute to the probe’s strong biocapture capabilities in an immobilized state on top the sensor platform leading to an overall better analytical performance.

### Pre-confirmation of E6 oncogene detection through PCR analysis

Before applying the designed sensor platform for the detection of HPV-16 E6 oncogene in cervical cancer gDNA samples, the performance of the designed E6 capture probe was tested through PCR analysis to determine whether the *in-sillico* confirmed target binding properties of the probe performs well under realistic and practical conditions. PCR amplifications act as a pre-confirmatory test, enabling us to deduce the E6 primer/probe’s target binding functionality and target selectivity using actual viral integrated human gDNA samples. Various parameters were optimized for both the PCR reaction as well as other earlier steps in order to obtain the best, reliable results regarding HPV-16 oncogene detection. Culturing of the HPV-16 positive and negative cell-lines were performed under highly stringent conditions with standard culturing procedures.

**Figure 4:**
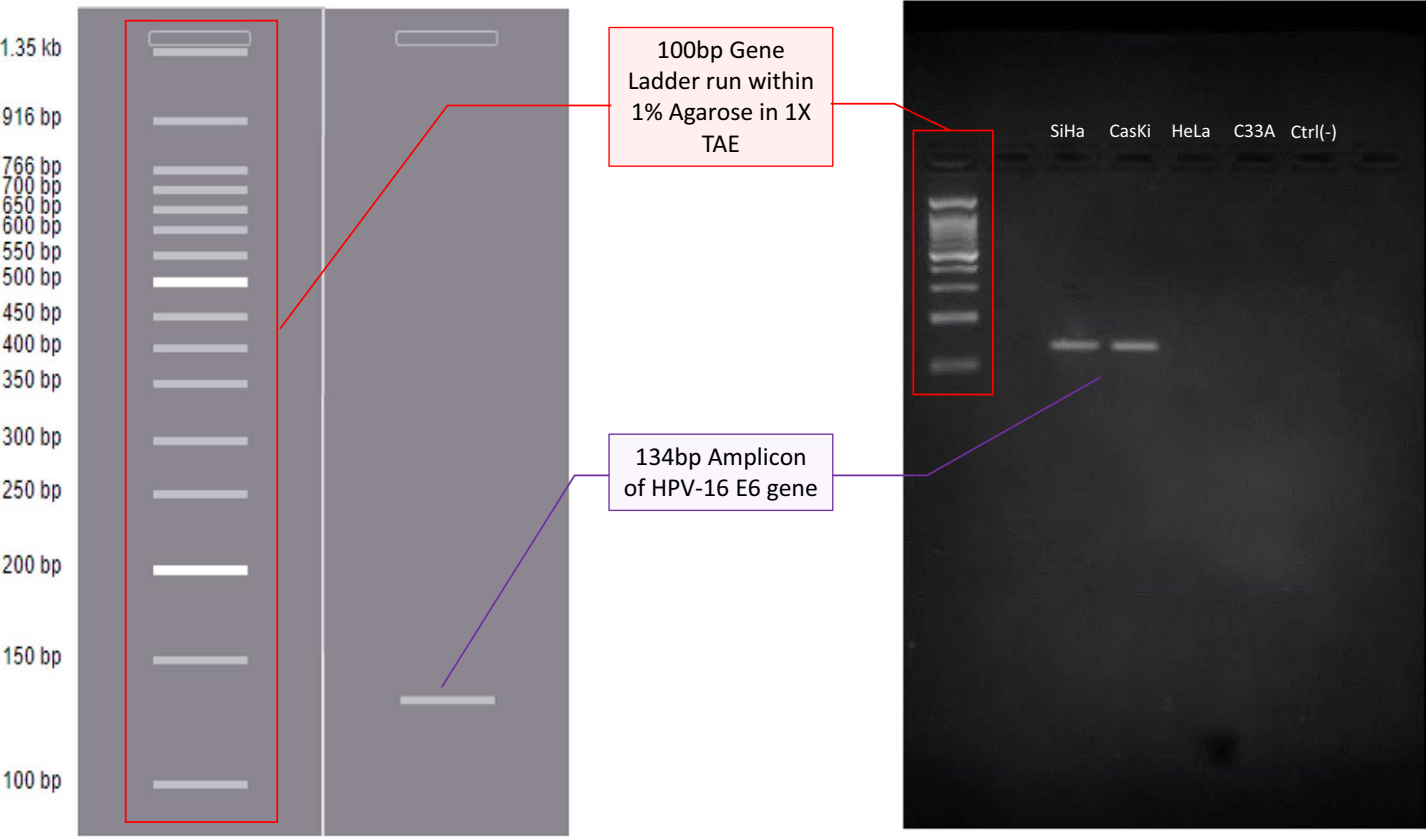
Diagram showing the comparison of virtual and experimental electrophoretic gel run of PCR products from E6 gene amplification. The left of the diagram shows virtual gel-run performed in GenomeCompiler software. The right of the diagram shows real gel image (full-length gel) run using 4 cervical cancer cell samples; SiHa, CasKi, HeLa, C33A and negative control lane on same gel.

PCR amplification for all four cell lines: SiHa, CasKi, HeLa, C33A and their respective gel electrophoresis results together with 1kbp DNA ladder are shown in Fig. 4. PCR analysis was in-fact carried out using 5′-thiolated primer sequences (forward primer; th-5′-AGCGACCCAGAAAGTTACC-3′, reverse primer; th-5′-GCATAAATCCCGAAAAGCAAAG-3′) which showed no disruption in the amplification of the targeted gDNA region but however thiolation at the 3′-end can noticeably lead to unsuccessful primer amplification. Gradient PCR runs were performed beforehand to optimize the annealing temperatures of the designed E6 primers. T_a_ of 55 °C provided the clearest band formation within a gradient range of 45–60 °C. A clear, defined band formation is present in both the HPV-16 positive cell lane (SiHa and CasKi) while no visible bands can be observed in the other two lanes having HPV-16 negative cells (HeLa and C33A). Furthermore, even though the HeLa cell-lines used during PCR test are positive for HPV-18 E6 viral DNA, a subtype of the same virus species, no such amplification bands can be recognized. This additionally fortifies the specificity of the designed E6 probe towards targeted HPV-16 E6 gene. The generated PCR results are highly accurate and reliable since the DNA bands in the gel run reflect the predicted amplicon band size and relative position seen the in virtual PCR-gel run, as discussed in the above section 3.1. Moreover, the PCR amplification results are in agreement with Wang Johanning* et al*., who have also obtained similar PCR amplicon bands for HPV-16 E6 gene regions in HPV-16 positive cell lines and portrayed no bands in HPV-16 negative cell lines^19^.

### Optimization of ZnO film for hydrothermal growth of ZnO-Nanorods

Several optimization steps were conducted to improve the ZnO film layer’s optical and electrical properties in order to ensure the growth of a quality-driven, conductive ZnO-Nanorods. Key parameters such as the choice of sol-gel solvents, stabilizer ratio, and film annealing temperature were optimized to enhance the ZnO transducer’s stability and structural crystallinity. Table 2 records the maximum transmittance values of ZnO sol-gels prepared with different MEA stabilizer ratios and sol-gel solvents. Stabilizer ratios lower than 1:1 exhibited higher transmittance compared to sol-gels having higher MEA to Zn ratio. Although ratios having greater transmittance values are preferred, in such cases using lower amounts of stabilizer leads to insufficient interaction between the ZnO nanoparticle and MEA resulting in uncontrollable nuclei growth and impaired film conductivity^20^, hence the reason of using 1:1 stabilizer ratio. On the other hand, from the comparison of different solvents used, IPA and ethanol portray a greater transmittance percentage compared to methanol. This is primarily due to methanol having a lower boiling point than IPA or ethanol, which causes rapid particle agglomeration and correspondingly a lower transmittance. However, ethanol is preferred since the use of IPA solvent is seen to produce a more viscous sol-gel that require longer stir time and higher amounts of stabilizers to properly condense the large, clumped ZnO particle until a clear sol-gel is obtained.

**Table 2:** Transmittance percentage of different solvents and stabilizer ratios.

Stabilizer Ratio	0.50:1	0.75:1	1.00:1	1.25:1	1.50:1
Transmittance	92%	50%	21%	1.2%	9%
**Solvents**	**Ethanol**	**Methanol**		**Isopropyl Alcohol**	
Transmittance	23%	3.7%		59%	

**Figure 5:**
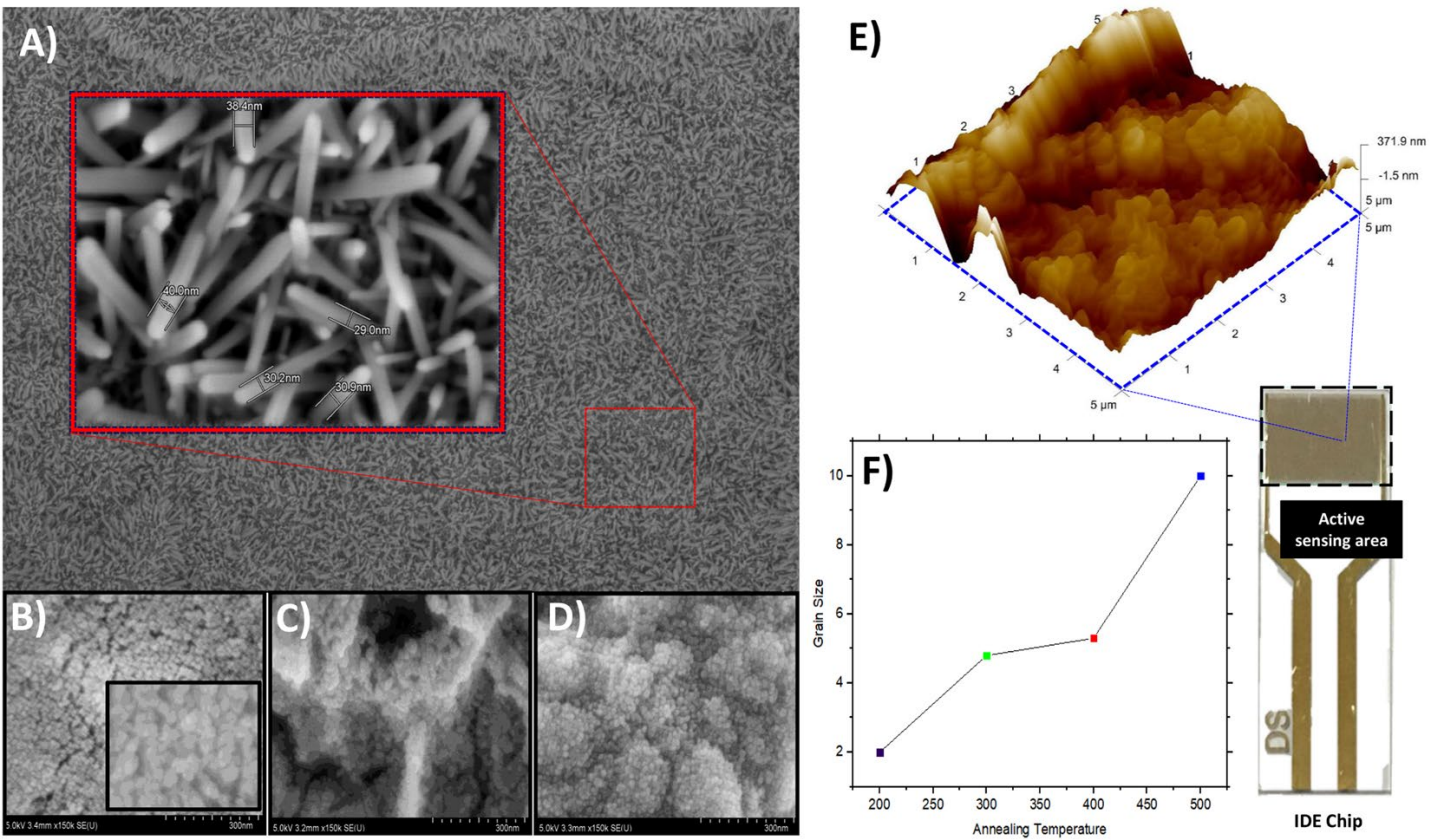
Diagram of morphological and surface analysis of ZnOAu-Nanorods layers. (**A**) FESEM images of hydrothermally grown ZnO-Nanorods. FESEM images of ZnO thin film using different solvents (**B**) ethanol, (**C**) methanol and (**D**) IPA. B insert shows a magnified FESEM image of ZnO grains in ethanol solvent. (**E**) 3D-AFM image of ZnO-Nanorods with the image of IDE chip’s active sensing area. (**F**) The plot of grain size against annealing temperature.

Furthermore, FESEM images of the three different solvents used for solvent optimization is also shown in Fig. 5(B–D). Overall, we can infer that all three solvents contributed a dense, uniform coating of circular ZnO particles and did not exhibit striking differences in ZnO film formations. The only difference observed is that the grains size exhibited in ethanol was much smaller compared to methanol and IPA since only at a higher magnification of 300 K can the circumferences of ZnO nanoparticles became clearer. ZnO grains were already visible around 150 K magnification in both IPA and methanol solvents. Such smaller sized ZnO grain formations in ethanol solvents further supported the choice of ethanol being the suitable solvent for ZnO-Nanorod synthesis since smaller sized grains can account for large total surface coverage. Hence, improving the electrical conductance of the ZnO-Nanorods by facilitating better grain to grain transfer of electrons^21^.

Optimization of annealing temperature of the ZnO films was additionally performed and analyzed using AFM analysis to choose the optimum temperature for ZnO grain crystallization. The plot in Fig. 5(F) illustrates the linear increment of grains size with annealing temperature. Such results could be contributed by the increased growth and crystallization of ZnO grains as well as the coalescence of adjacent grains crystals through atomic re-bonding at higher temperature ranges. Although higher temperature promotes better film crystallization, 300 °C was chosen as the suitable annealing temperature since it is the range at which resistivity of the films are reportedly much lesser due to the fact that metal oxide thin film crystallisations at higher temperatures result in lower electron charges or carriers that could profoundly decrease the grown Nanorods sensitivity and conductance properties as well as increasing dislocation density defects^22,23^. Low temperatures lead to improper grain crystallization, forming void/holes with the film’s structure and contributing to more structural defects. According to the experimentally obtained results, average surface roughness (RMS) values of film heat-treated at 300 °C is around 9 nm which was significantly higher than the RMS values of films treated at higher temperatures. This although contradicts the other previous reports on RMS value’s correlation with annealing temperature, it still reinforces the choice of annealing temperature since metal oxides film with a rough surface are stated to have large surface area to volume ratio and reduced electron scattering center effect which correspondence to improved biosensing applications^24,25^.

### Structural and morphological analysis of ZnOAu-Nanorods

Surface morphology of the ZnOAu-Nanorods observed form FESEM imaging, as shown in Fig. 5(A), indicate the definite formation of ZnO nanostructures. Precise and careful observation of the ZnO-Nanorods shows their diameter to be in the range of 30–40 nm in width and exhibit a tip shape very similar to hexagonal shaped structures. These protruding structures were shown to uniformly coat the entire surface without any visible pores or pin-hole formation which further consolidates the effectiveness of ZnO spin coating and hydrothermal growth processes. On a smaller scale of magnification, regions of contrasting color (not shown in figures) were perceivable which shows the formation of multiple layers of ZnO-Nanorods. This, in fact, could also be due to ZnO nanoparticle agglomeration within the prepared ZnO sol-gels prior to the coating procedure. A noticeable feature in Fig. 5(A) is the lack of a clear depiction of AuNP’s present on top of the ZnO-Nanorods. Theoretically, this could be a distinctive attribute of the different AuNP deposition technique employed in our methodology relative to sputtering forms of depositions. However, this different approach of AuNP doping did not reduce their chemisorption properties of organic molecules such as DNA/RNA molecules during immobilization and hybridization processes as seen in the following analysis.

The surface topology was further assessed through AFM analysis. Figure 5(E) presents a 3D depiction of the hydrothermally grown ZnO-Nanorods. Although the image does not portray the defined shapes of the ZnO-Nanorods as seen in FESEM analysis, the hexagonal shaped tips of the Nanorods are still recognizable. Such low image clarity could be caused due to the growth direction of ZnO-Nanorods being skewed towards a more lateral incline instead of a more vertical growth axis. Since AFM analysis principally uses the interaction of sharped edged cantilevers with the sample surface and registers the change in force and motion of the probe to map the surface topology of samples, Nanorods grown below an angle of 90° to the substrate base could affect or reduce the probe’s interaction with their inter-rod spacing, resulting in images with low resolution of the actual ZnO-Nanorods definite shapes. This inference is supported by similar AFM image results of ZnO-Nanorods obtained by Khodair *et al*.^26^. RMS values of ZnO-Nanorods were also confirmed to be 99.2 nm through AFM analysis.

**Figure 6:**
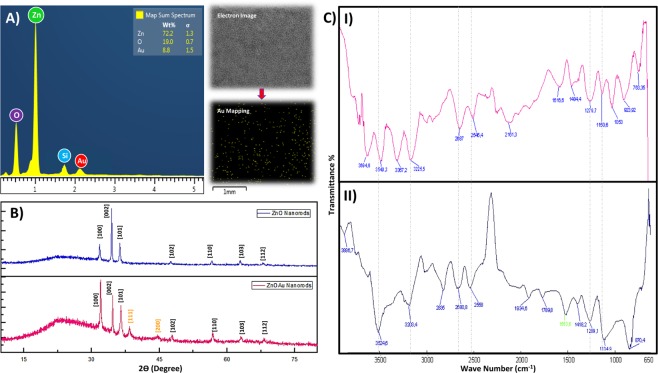
Diagram of structural analysis of ZnOAu-Nanorods. (**A**) EDX spectrum of Au doped ZnO-Nanorods. Left of (**A**) shows the map image of AuNP deposited on top of ZnO-Nanorods. (**B**) XRD peaks of doped and undoped ZnO-Nanorods. (**C**) FTIR spectrum of nanorod layers with E6 probe immobilized (I) and target hybridized (II).

Structural analysis of the ZnOAu-Nanorods was performed through XRD characterization and EDX-based elemental analysis. XRD analysis was conducted to determine the crystal quality, crystallite size as well as their preferential growth orientation. Figure 6(B) depicts the XRD data obtained for both Au doped and undoped ZnO-Nanorods. Simply through observation of the multiple diffraction peaks obtained, we can spot the difference between ZnO matrixes with and without AuNP’s. These diffraction peaks are in good accordance with the reference peaks shown in JCPDS cards for ZnO and Au respectively. The ZnO-Nanorods are said to be in the wurtzite phases and exhibit excellent crystallinity since their XRD spectrum produce sharp, narrow peaks at 31.02°(100), 34.40°(002), 36.31°(101), 47.65°(102), 56.65°(110), 62.89°(103) and 68.00°(112). Similarly, the diffraction peaks at 38°(111) and 44°(200) were assigned to peaks that reflect face-centered cubic of AuNP’s ^27,28^.

Based on XRD peaks of undoped ZnO-Nanorod, (002) was the peak that held the highest intensity, demonstrating to be the dominant plane of growth orientation and hence was used to perform further computations regarding the matrix’s structural and crystallite attributes^24,25^. The following formulae are utilized, where λ represent the X-ray wavelength of the incident Cu K˛ radiation (0.154056 nm), α and c are the lattice constants, D is the crystallite size and *δ* is the dislocation density defect.

1 $${a}=\sqrt{\frac{1}{3}\frac{{\lambda }}{{\sin }\,{\rm{\theta }}}}$$

2 $${c}=\frac{{\lambda }}{{\sin }\,{\rm{\theta }}}$$

3 $${\delta }=\frac{1}{{{D}}^{2}}$$

By computing the c and a-axis of the ZnO matrixes using Eqs (1) and (2), we could see that the lattice parameters ‘c’ is relatively greater than ‘a’. This conclusively states that the fabricated ZnO-Nanorods exists as pure hexagonal wurtzite structures and possess a preferred c-axis orientation. The crystallite sizes of ZnO were also determined using Scherrer equations to be 52 nm which correspondence to values obtained by previous reports^24,^^28^. The values also correlate well with the average grain size determined from AFM analysis (51.6 nm). Following doping of AuNP, the XRD spectrum of the composite matrix yields a lower peak intensity of (002) and a shift in intensity towards lower angles 31.02°(100). This could possibly be an effect of embedding of AuNP into the ZnO matrix which induces strains that disorders the d-interspacing between the ZnO crystal lattice, leading to constraints in crystallites growth^29–31^. These are evidently seen when the calculated crystallite size of doped ZnO reduces to 40 nm which indicates the existences of a dopant-induced compressive stress^31,32^. The lattice constants were also observed to increase for the doped matrix compared to undoped Nanorods as shown in Table 3. The crystallite size of AuNP was also similarly determined using Scherrer equations to be 17.18 nm.

**Table 3:** XRD peaks information and crystal parameters.

Samples	XRD peaks	FWHM	Crystallite size (nm)	c constant	a constant	Dislocation density defect (*D*)
ZnO-Nanorods	34.42	0.1596	52.12	0.52	0.42	0.00037
Au Doped ZnO-Nanorods	31.98	0.2055	40.21	0.56	0.43	0.00062

To further assess the crystalline quality of the grown ZnO-Nanorods, dislocation density was determined using Eq. (3). The values calculated for both doped and undoped ZnO-Nanorods presented a very small value at 10^−4^, signifying the compactness of the grown matrix with lesser structural defects^33^. Such improved integrity of the matrix testifies the reason for ZnO film optimization before the growth of Nanorods since the proper choice of important factors that influence grains size and quality such as stabilizer ratio, solvent and optimum temperature for film annealing produces the grains size that muffles structural defects but simultaneously maintains film conductivity. These values increase slightly by two units for the Au doped ZnO matrix which in fact is a natural event given the presence of a metal impurity and thus be considered as a non-factor. The obtained EDX spectrum shown in Fig. 6(A) for the ZnOAu-Nanorods supportively concludes both the presence of AuNP’s within the pure ZnO matrix and the absence of other impurities. Additionally, the uniform distribution of AuNP’s over the ZnO-Nanorod layers observable form the Au-mapping seen in Fig. 6(A) implies the effectiveness of the deposition techniques used.

### Effect of dopant and counteractants on the performance of ZnO-Nanorods

**Figure 7:**
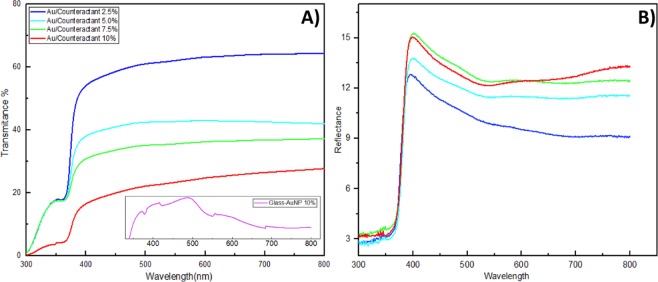
Transmittance (**A**) and Reflectance (**B**) spectrum of ZnO-Nanorods with varying concentrations of AuNP and citrate counteractants. (**A**) Insert shows transmittance spectra of glass-coated with AuNP at 10%.

Varying sizes of metal oxide nanoparticles and dopant concentrations are known to affect both the optical and optoelectronic performances of composite transducer layers. In order to analyze these influences, the combinatorial effect of increased sizes of ZnO nanoparticles (due to the addition of increased citrate counteractants) and increased concentrations of Au dopants (2.5%, 5%, 7.5%, and 10%) were analysed through UV-Vis spectroscopy within a scan range of 300 to 800 nm wavelength. From Fig. 7(A,B), we can observe that all the doped nanostructured films possessed transmittance below 65% and smaller reflectance properties due to their dense layer of grown ZnO-Nanorods. All nanostructured films produced a peak in transmittance around 360-370 nm indicating the presence of crystalline ZnO grains. Presence of larger ZnO nanoparticles and addition of higher concentrations of AuNP’s was seen to continuously reduce the transmittance properties while increasing the reflectance of the films. This is speculated to be a coupled light scattering attribute of both large AuNP’s^34,35^ and an increased size of ZnO nanoparticles consequential of increasing amounts of citrate counteractants. Larger diameter ZnO nanoparticles coupled with an increased thickness of gold nanoparticle layer over the Nanorods at higher AuNP concentrations lead to greater optical scattering effect and hence lower transmittance quality.

It is well known that the presence of AuNP’s in any film matrix results in the occurrences of local surface plasmon resonance (LSPR) leading to a characteristic transmission band within the range of 500–600 nm^36^. Intriguingly even at higher concentrations of AuNP’s, the absorption curves apparent for gold nanoparticles at a range of 400–600 nm which signifies their plasmonic absorption properties is absent within the optical spectra of ZnOAu-Nanorods as seen in Fig. 7, where only a constant transmittance signal is observed beyond 400 nm. To investigate such inferences, we analyzed the transmission spectra for glass substrates deposited with only 10%-AuNP’s and no ZnO-Nanorods. The spectra obtained (shown in Fig. 7 insert) clearly depicts the presence of an absorption band which can be seen from the subtle decrease in transmittance values in the expected range of 500–600 nm, indicating the absorption of electromagnetic wavelengths and the subsequent plasmonic resonances upon irradiation^37,38^. Although this demonstrates to a certain level the existence of a masking effect of ZnO-Nanorods over the plasmon excitation bands of AuNP’s, the mechanisms behind such phenomenon is unknown. The transmittance spectra of the 10%-AuNP also confirm the spherical shapes of gold nanoparticles due to the absence of multiple absorption peaks^36,39^.

4 $${\alpha }=\frac{{ln}(1/{T})}{{d}}$$

5 $${\alpha }\mathrm{hv}={\beta }{({hv}-{Eg})}^{1/2}$$

**Figure 8:**
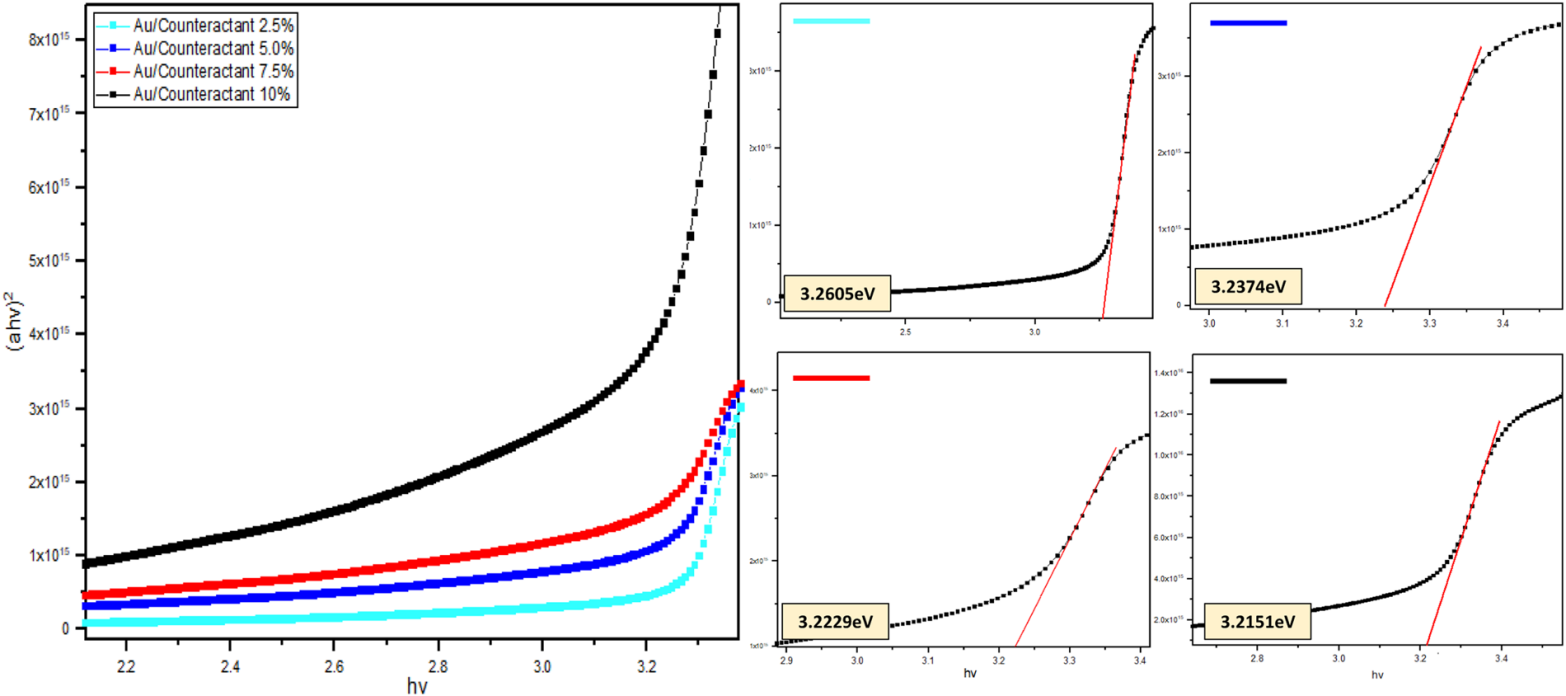
Diagram of Tauc plot (*αhv*)^2^ vs *hv* and respective band gap energy values (eV).

The optical energy bandgap (E_g_) of Nanorod layers were determined using Eqs (4) and (5) to calculate the absorption coefficient, α and subsequent derivation of the Tauc plot (*hv* vs (*αhv*)^2^) illustrated in Fig. 8. *d* represents film thickness, T is the transmittance value, *v* represents the frequency of the incident radiation, *h* is the plank’s constant and 1/2 is referred as the direct allowed transition associated for electronic transitions. Extrapolation of each plot’s linear portion allows elucidation of the E_g_ displayed in Fig. 8. As expected, the band gaps for the ZnO-Nanorods showed a consistent decrement in accordance with increasing counteractant and dopant concentrations. Such a phenomenon is contributed by two main factors; reduced quantum confinement effect combined with strong Au to ZnO interfacial coupling. Since both the d and p orbits of gold and oxygen atoms have t2 symmetry in a tetrahedral configuration, excellent p-d coupling occurs when AuNP occupy defect sites within the ZnO matrix. This orbital hybridization leads to oxygen 2p levels being moved up and subsequent narrowing of ZnO energy band gaps^36,40^. On the other hand, due to prior treatment of ZnO sol-gels with increasing amounts of citrate counteractants, the interaction of the citrate ions with MEA neutralizes the stabilizer’s charges which in turn contribute to nanoparticle agglomeration. Thus, the resulting larger sized ZnO nanoparticles curbs the influence of quantum confinement effects^24,41,42^.

6 $${{E}}_{({gap},{nanocrystal})}={{E}}_{({gap},{bulk})}+\frac{{\pi }^{2}{{h}}^{2}}{2{{R}}^{2}}(\frac{1}{{{m}}_{{e}}}+\frac{1}{{{m}}_{{h}}})-0.248{{E}}_{{Ry}}$$

This is further mathematically proven in Eq. (6), which governs the relation of grains size with quantum size effect and bandgap, where R is the grain radius, *h* being planks constant, *m* representing hole effective mass and E_Ry_ being bulk binding energy. Here, we can infer that the increase in crystal radius mathematically reflects a reduction in bandgap values^43^. Elimination of quantum size effects on ZnO-Nanorods promotes improved film conductivity owing to increased electron movement since the constraints acting upon the electrons within small crystallites having greater grain boundaries have been reduced. Although ZnO grains with increased sizes produce smaller band gap values, excessive particle aggregation can equally lead to lower film optical quality and conductivity^44,45^. However, these issues are circumvented with the addition of Au dopant since the presence of such nanometals provide constraints on grain enlargement, resulting in a controlled form of grain coalescences. This correlates well with both the XRD ad AFM results, where the grains size for doped ZnO-Nanorods are significantly smaller compared to counteractant treated, undoped film. The coalition between both factors of Au doping and controlled ZnO grain growth demonstrate promising band gap energy values comparable to those E_g_ values from previous reports^24,28^ utilized both the factors separately. This indicates that both variables can be used in unison to contribute to a better semiconductor optical and conductive properties.

7 $${\alpha }={{\alpha }}_{{o}}{\exp }(\frac{{hv}}{{{E}}_{{u}}})$$

8 $${n}=(\frac{1+{R}}{1-{R}})+\sqrt{\frac{4{R}}{{(1-{R})}^{2}}}-{{k}}^{2},\,{k}=\frac{{\alpha }{\lambda }}{4\pi }$$

**Figure 9:**
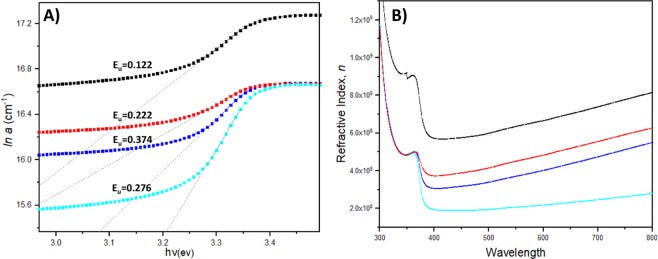
(**A**) Diagram of the relation of Urbach energy with photon energy and absorption coefficient. (**B**) The plot of refractive index against wavelength.

Based on the absorption coefficient calculated, the graph of *ln α* vs *hv* was plotted as shown in Fig. 9, to study the disorder caused by band tail formation due to Au doping^46,47^. Using the slope of the linear portion of the plot, Urbach energy (E_u_) was determined based on Eq. (7) which governs the dependence of *α* on photon energy near the conduction band edge^48,49^. The increase in E_u_ values shown in Fig. 9(A) indicates that the disorder within the film continues to increase as well^50,51^. However, a slight variation was seen where the E_u_ values decrease at higher doping concentration and ZnO particles size, stipulating better Nanorod quality and relatively lower structural defects. Furthermore, Fig. 9(B) also shows the interrelation between wavelength and refractive index (*n*) of the ZnO-Nanorods using Eq. (8), dependent on transmittance, reflectance, R and extinction coefficient, *k*. Refractive index of the Nanorod film holds an inverse relation with the wavelength while exhibiting a linear relation with dopant and counteractant concentrations. Au doping leads to greater intensities of optical energy that serves as the center for light ray dispersion which in turn correspondences to increased reflectance properties at higher dopant concentration^52^. Improved grains size at higher counteractant concentration further augments the increment in refractive index since larger grains size contributes to lower oxygen vacancies and free carrier density^53^. Both E_u_ and *n* precisely indicate the functional benefits of incorporating both grain size and dopant concentration factors in fabricating better metal oxide films and nanostructures.

### Preliminary verification of ZnOAu-Nanorod biosensing properties

Biosensing capabilities of the tailored ZnOAu-Nanorods were confirmed through FTIR spectroscopy to analyze the presence of different functional groups and bond stretching occurring during E6 probe immobilization and targeted gDNA hybridization. Fig. 6(C-(I,II)) shows the FTIR spectra for both probe immobilization and after target hybridization. From the spectrum of both immobilization and hybridization, multiple absorption peaks were registered, specifically at regions 800–1700 nm indicating the presence of groups and bonds of immobilized nucleic acid moieties, the fingerprint region for DNA molecules^54^. Absorption peaks at 870 cm^−1^ and 923 cm^−1^ within both spectrums represent the deoxyribose phosphate backbone of ssDNA strands^55^. Absorption peaks at 1418, 1484, 1616, and 1789 cm^−1^ in both spectra correspond to the functional bases found in DNA strands such as cytosine, thymine, and guanine^56^. Vibrational peaks at 1053, 1134, 1150, 1279 and 1289 cm^−1^ hint the occurrence of symmetric and asymmetric phosphate group and phosphodiester bond stretching ^55,56,57^. Spectral regions between 3200–3800 cm^−1^ indicate the presence of N-H stretching vibration of purine and pyrimidines rings in DNA bases^58,59^. Hybridization of gDNA with immobilized probes was confirmed from the increase in vibrational peak intensity compared to the peaks observed for the immobilized spectrum including additional characteristic peaks at regions between 800–1600 nm and 2500–3800 nm. Such results clearly suggest the success of probe immobilization and ascertain the biocapture properties of the probed ZnOAu-Nanorods.

### HPV-16 detection analysis using impedance spectroscopy

**Figure 10:**
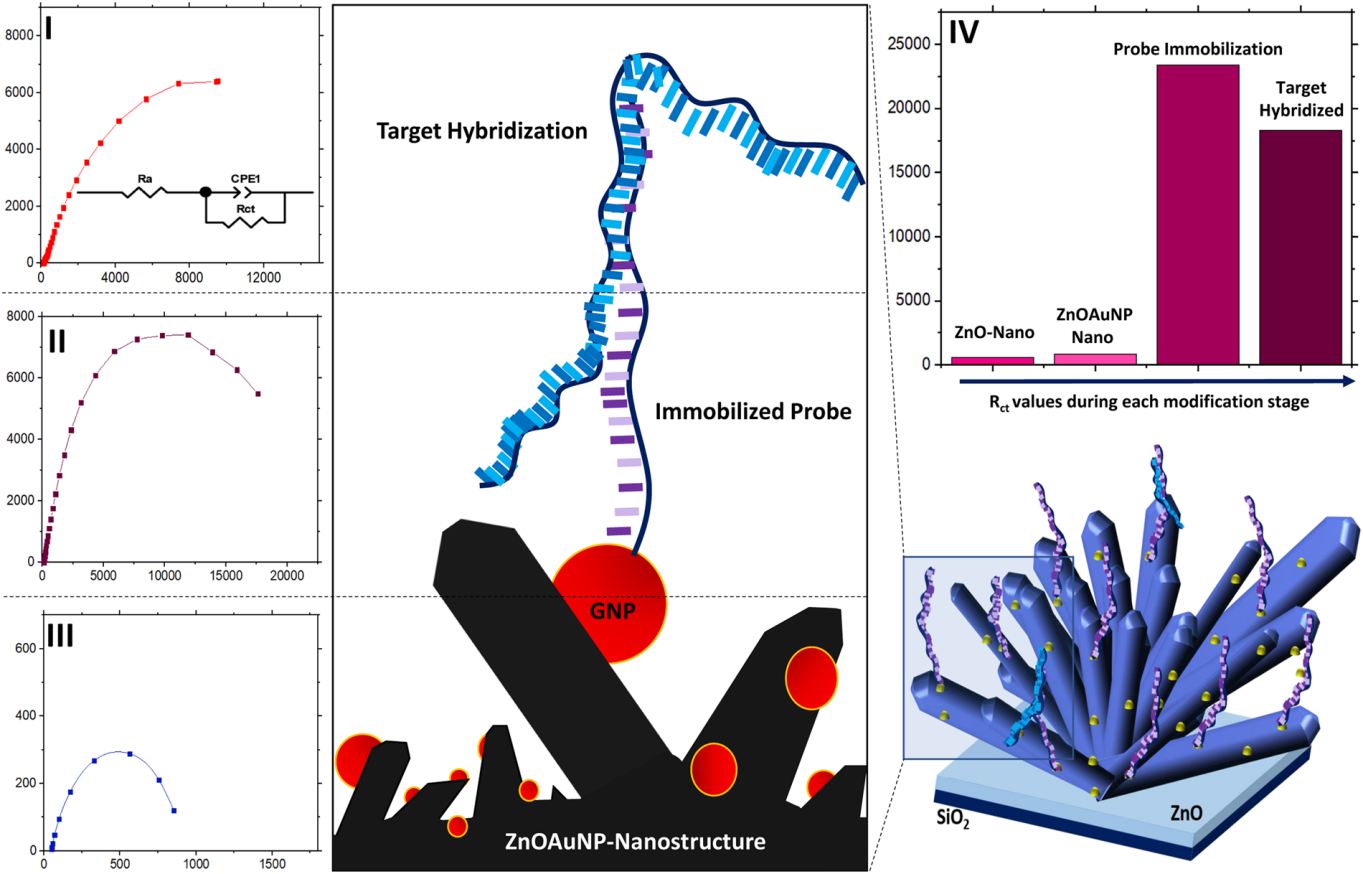
Diagram of measured EIS signals at each stage of chip modifications; (I) growth of ZnOAu-Nanorods, (II) during probe immobilization and (III) target hybridization. (IV) Shows a bar chart of calculated R_ct_ values against the stages of modification.

The electrochemical and surface charge properties of the ZnOAuNP-Nanostructure/E6-probe modified IDE biosensors for HPV-16 E6 oncogene detection was characterized using electrochemical impedance spectroscopy (EIS) in the presence of PBS solution mixed with 2 mM of K_3_(Fe(CN)_6_)/K_4_(Fe(CN)_6_). The obtained Nyquist plot from each of the EIS measurement was fitted with Randles equivalent circuit, enabling subsequent derivation of electrical parameters such as bulk solution resistance (R_a_), charge transfer resistance (R_ct_) and constant phase element (CPE). Each parameter has in its own independent significances which helps accurately model characteristics of the electrochemical impedance experiment, especially at the probe/target hybridization interfacial layer. For instance, double-layer capacitance CPE was chosen as a curve fitting parameter instead of simple, pure capacitance since it can account any surface inhomogeneity caused due to doping of AuNP into the ZnO, thus subsequently circumventing reduced surface uniformity and variation in the signal relaxation time^28,60,61^. The parameter interested in studying biomolecular interactions, in this case, is the R_ct_ value which equals the semi-circular diameter of each EIS measurement plot. Such R_ct_ values represent the resistance against the electron-transfer potential of the redox probe, ferricyanide in the surrounding droplet of electrolyte solution towards the probe immobilized on the surface of the modified electrodes. Changes in the surface dependent R_ct_ values during each stage of chip modification, including probe immobilization and target hybridization, is well portrayed in the obtained impedance spectrum, presented in Fig. 10(I–IV).

As inferred from the above diagram, the EIS spectrum corresponding to the IDE sensor coated with ZnO-Nanorods (without AuNP dopants) yielded R_ct_ values around ~600 Ω (>91 Ω of ZnO films). An increase in the R_ct_ values is apparent for the spectrum obtained from ZnOAuNP-Nanorod which registers around ~850 Ω. This increase in resistance can be explained in terms of n and p-type semiconductor interactions at a quantum level. ZnO matrix alone has a very low charge transfer resistance but however adsorption of oxygen atoms at the ZnO surface during processes such as annealing creates a current/flow of electrons from the ZnO matrix towards the O_2_ atoms, causing the ZnO layers to harbor greater resistance. These O_2_ entrapped electrons induce spatial charge formations which create local electric fields that further impede film conductivity^62^. Larger surface area to volume ratio of hydrothermally grown ZnO Nanorods causes even greater resistance since it leads to an increased amount of surface adsorbed O_2_ atoms and subsequent greater restriction in the movement of spatially trapped conduction band electrons. This explains why ZnO Nanorods yield slightly higher R_ct_ values compared to bare ZnO thin films as shown in Fig. 10(IV). Doping of AuNP’s into the ZnO matrix further augments R_ct_ values since each Au atoms exist in higher Fermi energy levels owing to their lower work function^28,^^63^, resulting in the flow of electrons from Au to the ZnO layer. This electronic transition to the ZnO layers possessing lower conductivity creates pn-junctions at the Au/ZnO interface. Formation of such ohmic junctions coupled with negative repulsion of citrate-stabilized AuNP’s against the redox probe could consequently attribute towards greater charge transfer resistance^64^.

During probe immobilization, the interaction of the DNA probe with the ZnOAuNP-nanostructure’s surface, mediated through SH-Au bond formation gives rise to an exponential increase in R_ct_ values, recorded around ~23400 Ω. This is because the negatively charged phosphate backbone of immobilized E6 probes create a layer of accumulated negative charges in close proximity with the ZnO surface. This consequentially induces a strong electrostatic repulsion against the redox charges leading to the observed higher R_ct_ values. Utilization of electrons from Au/ZnO exchange for SH-Au covalent bond formation can cause broadening of the depletion layers and additionally lead to a significant increase of R_ct_ values as well^28,65^. In contrast to this, a slightly lower R_ct_ value was observed during gDNA target hybridization which was around ~9100–19500 Ω. We speculate that Manning’s theory of counterion condensation could play a role in such conditions. Since gDNA is basically a long polyanionic rod spanning up to 3.2 million bases of negative phosphate molecules, monovalent ions in the PBS buffer and already existing other di/trivalent ions present within the extracted gDNA sample could very well contribute to counterions condensation along the gDNA’s length, resulting in counterion-induced negative charge neutralization^66–68^. Besides this, natural compaction of the large gDNA strand into a denser, packed structure (resultant of counterion condensation) under *in-vitro* conditions and low-temperature induced strand folding during hybridization steps could likewise also contribute to lower negative potential^69,70^. Such factors can cumulatively cause the net charge of the gDNA to switch from negative to slightly positive, creating a counter-balance effect against the negatively charged probe layer during probe/target association. Therefore, negating the coulombic repulsion exhibited by the probe layer against diffusion of redox ions towards the electrode surface and leading to an overall lower R_ct_ value including the observed downwards shift in the impedance spectrum during target hybridization.

### Assessment of the HPV-16 sensor’s analytical performance

**Figure 11:**
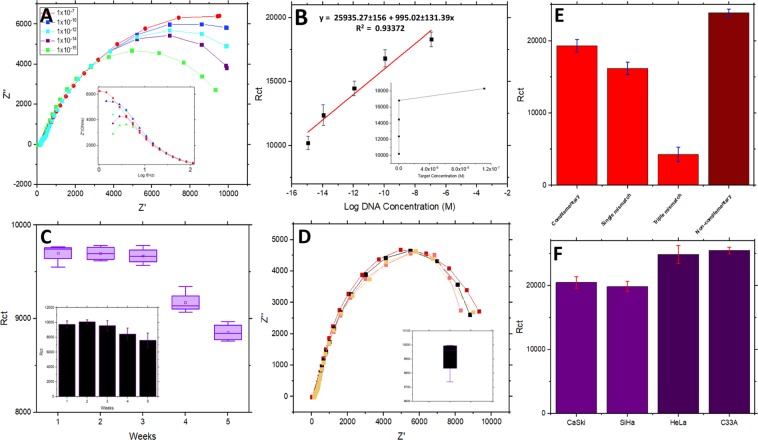
(**A**) Depicts the EIS signals obtained at each different concentration of target HPV viral sequence and (**A**) insert showing the plot of Z” values against log frequency (Hz) for the different target concentrations. (**B**) Shows the plot of R_ct_ values against Log gDNA concentrations and (**B**) insert showing the plot/curve of R_ct_ values against molar target concentrations. (**C**) Shows the stability of modified IDE sensors tested over 5 weeks. (**D**) Shows the reproducibility of the sensor platform taken from 4 different sensor replicates. Bar chart representing R_ct_ value measured for different target sequences (**E**) and clinical cancer cell samples (**F**).

The modified sensor’s analytical parameters were tested, and the results are interpreted accordingly, as presented in Fig. 11(A–F). From Fig. 11(B) insert, we can observe that the plot of R_ct_ vs target DNA concentrations follows a curve pattern similar to Langmuir adsorption curve. Since platforms with such electrochemical features require more complex fitting functions to estimate an accurate limit of detection (LOD) value, derivation of LOD through a linear fit function using 3σ is less reliable and inaccurate. Hence, the sensor’s actual current response or R_ct_ values for the lowest concentration of gDNA integrated viral target functionally distinguishable from the blank/probe signal was used to accurately evaluate the LOD values, which is also correspondingly reported by other authors^71,72^. A graph of R_ct_ values against Log (DNA concentrations) was plotted to evaluate the sensitivity and linearity of the modified IDE sensor. The sensor displayed good linearity indicated by r^2^ values equivalent to ~0.93 and high levels of sensitivity indicated by their detection limit of 1fM. In the previous section, it was explained in detail regarding the reason behind the observed decrease in R_ct_ values between immobilization and target hybridization. During sensitivity measurements, the typical electrochemical impedance pattern was visible, where the R_ct_ values were observed to linearly increase as the targeted gDNA concentration increases. This is due to the fact that at higher gDNA concentrations, a greater number of gDNA strands can be found hybridized to the E6 capture probe, increasingly populating the electrode surface. This can possibly lead to higher degrees of steric hindrances that obstruct the accessibility of the electrode’s surface area for the redox ions and consecutively increasing R_ct_ values. For further affirmation regarding the sensitivity of the HPV-16 sensor, each obtained R_ct_ values of different gDNA concentrations were computed with t-test analysis. The p-values obtained between each consecutive measurement was <0.05*α* which statistically proves the significant difference between each different EIS measurements. On another note, the plot of the imaginary part (Z”) against the log frequency manifested the same pattern seen during sensitivity measurements in Fig. 11 (B insert). A gradual increase in the imaginary part at higher gDNA concentration dictates the reduced conductivity of the modified layers.

Specificity assessments were conducted using different cell-lines previously employed during PCR pre-confirmatory test as well as other altered viral E6 gene sequences, which included single base-mismatch, triple base-mismatch, and non-complementary strands. As shown in Fig. 11(E,F), R_ct_ values recorded for the complementary E6 gene target was the highest among the other samples while a 72% and 20% lower signal difference was observed for the R_ct_ values of triple and single mismatch strands respectively. A similar result was obtained for the cell-line samples in which both SiHa and CasKi cells positive for HPV-16 exhibited near-identical R_ct_ values recorded for the complementary E6 strand. On the other hand, the non-complementary strands, HeLa and C33A cell-lines (HPV-16 negative) showed non-relevant R_ct_ values since the registered resistance was highly similar the signal recorded during probe immobilization, indicating both the absence of target hybridization and excellent selective properties of the designed sensor platform. These results correlate accurately with the PCR amplification results conducted earlier as well, thus demonstrating the performance of the designed E6 capture probe under free and immobilized conditions. Reproducibility and stability tests conducted using 1 nM of gDNA also displayed favorable results. The sensors reproducibility was observed to be fairly good since it yielded a standard deviation (STD) of 4.2% among the 4 concurrently obtained readings, prepared and measured under the same experimental conditions. The sensor also possessed excellent, long-term stability since the R_ct_ values were only reduced by 10–25% even during the 5^th^ week of measurement, kept under 4 °C storage conditions.

## Methodology

### Primer designing and *In-silico* pre-analysis

The HPV-16 E6 oncogene sequence is obtained from NCBI in FASTA format. Using the sequences as the template, 19–22-mer forward and reverse primer sequences were designed targeting specifically the E6 oncogene using online primer designing tool, Primer3 and were subsequently ordered from Integrated DNA Technology.Inc with thiol modifications at 5′-end. Primer’s sequence and information are summarized in Table 4. The primer sequences are subjected to various *in-silico* based assessments. Secondary structure formations for the primers were analyzed using the web-based Mfold software (http://mfold.rna.albany.edu). Further analysis was performed to evaluate their binding probabilities. Virtual PCR runs were also performed using GenomeCompiler tool to analyze the binding specificity of the designed primers against the targeted E6 genes. HNADOCK webserver with PyMol visualization software was used to analysis the E6 probe’s binding probabilities, energetics, and secondary structure surface properties.

**Table 4:** E6 primers sequence length, GC content and annealing temperature.

Label	Sequence	Length	Tm	GC%
Forward sequence	5′-AGCGACCCAGAAAGTTACC-3′	19	51.3	52.6
Reverse sequence	5′-GCATAAATCCCGAAAAGCAAAG-3′	22	51.2	40.9

### Genomic DNA (gDNA) extractions from cultured cervical cancer cells

Cervical cancer cells were cultured under stringent conditions as per the instructions given by ATCC. Once the CO_2_ incubated cancer cell lines reach a confluency of more than 80%, they are subjected to gDNA extraction. The chosen cervical cell lines, *CaSki* and other viral infected cell lines such as HeLa, SiHa and C33A, which are adherent in nature, are harvested by trypsinization. Trypsin-EDTA solution (ATCC® No. 30–2101), balanced salt solution [Dulbecco’s Phosphate Buffered Saline without calcium or magnesium, ATCC® No. 30-2200], and complete Dulbecco’s modified Eagle’s medium (DMEM) with 10% fetal bovine serum and 1% streptomycin solutions are prepared beforehand. The cell culture medium within culture flasks is removed and added with 2/3 ml of trypsin-EDTA followed by rinsing with PBS solution and incubation at 37 °C for 5 mins. The detached cells are added with PBS solution to inactivate trypsin residues and transferred into 15 ml falcon tube. The cells are then pelleted down at 1000 rpm for 10 mins. The pellet is resuspended in 2 ml of 1X RIPA lysis buffer and vortexed well to lysis the collected cells. These cells are then sonicated 50 Hz for a few seconds and place them into ice container for 1 min. Sonication will be done repeatedly for 3 times. The lysed cells can be kept in −20 °C freezer for DNA extractions.

Phenol:chloroform:isoamyl alcohol (PCA) solution is prepared with a ratio of 25:24:1. Lysed cells are portioned into smaller volumes and transferred into 2 ml microcentrifuge tubes. Each tube is mixed with equal volumes of PCA solution, which is then properly mixed by inverting the tube several times. The tubes are then subjected to centrifugation at 13000 rpm for 15 mins, separating the solution into aqueous and organic phases. gDNA of cervical cancer cells are obtained by carefully withdrawing/pipetting the top clear, aqueous portion within the tube. These steps are repeated one more time and two more times with chloroform instead of PCA solutions. The obtained aqueous solution containing extracted cancer cell genomic DNA is then ethanol precipitated by adding 2X of 99% pure ethanol and 0.01X of NaCl solution to each tube. The solution is mixed gently for about 10–20 times followed through with centrifugation at 13000 rpm, 15 mins. After centrifugation again, the ethanol is removed and discarded where the pellet of DNA is then resuspended in 100 ml of TE buffer.

### PCR amplification confirmatory tests

PCR amplification is performed using a mix of 25 μl of DreamTaq Green PCR master mix purchased from ThermoFisher Scientific, 3 μl of 10 μM custom made forward (F) and reverse (R) E6 primers each, 15 μl of nuclease-free water, and 4 μl of 100–200 ng/ul of extracted gDNA, at 35 cycles using BioRad S1000 Thermocycler system. PCR cycles begin at 95 °C for 3 mins the followed by 30cycles of denaturation at 95 °C for 30 s, annealing at 55 °C for 30 s and Extension at 72 °C for 30 s. The resulting amplicons are analyzed using agarose gel electrophoresis methods with 2% agarose gel, 1X Tris-Acetate-EDTA (TAE) buffer and Ethidium Bromide stains, run in a horizontal electrophoresis chamber system from BioRad at 100 V for 1 hrs. The gel run is analyzed by using BioRad Universal Hood II chambers. The amplified bands for each sample are observed and the UV image of the gel is captured and visualized.

### Preparations of ZnO Sol-Gel solution

The Zinc acetate of 1.756 g is measured and added to 40 ml of molecular grade ethanol solution in a reagent bottle. The mixture is heated up to 60 °C and mixed at ~1000 rpm using magnetic heat stirrer until 20 mins. Then for the next 2 hrs, monoethanolamine (MEA) of 40.7 μl (stabilizer substances, maintained at 1:1 to the Zn Acetate) is added at a time interval of 10 mins (total 12 times) until the volume of monoethanolamine totals up to 488 μl while the solution is in a constant stirring until a clear, transparent solution is obtained. The sol-gel is then left to cool/age in a dark area for at least 2–3 days. Citrate ions (MEA stabilizer counteractants) were added at varying percentages (2.5%, 5%, 7.5% and 10%). Clumped residues were removed by centrifuging at low RPM speeds with subsequent removal of supernatant sol-gels.

### Coating of ZnO thin films on IDE chips

The interdigitated electrodes (IDE) chips are purchased from SilTerra Malaysia Sdn.Bhd. The chips are washed gently with acetone and quickly rinsed with distilled water to remove dirt particles. The prepared ZnO Sol-Gel is spin-coated onto the chips sensing surface area using Laurell WS-650-Hzb-23B model spin coater. The initial spin speed is at 700 rpm for 20 s followed by increasing spin speed to 3000 rpm,30 s and spin stop for 5 s to allow the layers to settle from the rotational movements. The wafers are then heated at 60–150 °C for 10–30 mins to remove or evaporate remaining solvent used. The coating process is repeated another two times to allow a total of three standard coating layer. The chips are then subjected to proper annealing at 300 °C for 2 hrs which will allow high crystallization of the ZnO film layers. Optimization steps of the ZnO thin films were carried out, specifically for the sol-gel solvent, stabilizer ratio, and annealing temperature, prior to nanorod growth.

### Hydrothermal growth of ZnO Nanorods and doping with AuNP

ZnO-Nanorods are grown using hydrothermal growth methods described in *Perumal et al*.^28^. Zinc nitrate hexahydrate and hexamethyltetramine are weight to 1.487 g and 0.701 g respectively to obtain 0.025 M of both substances (1:1). Both chemicals are mixed in distilled water of 400 ml (200 ml each) and stirred at ~1000 rpm at RT for 20 mins. The ZnO film coated chips are then dipped into this mixed solution. The solutions with the chip’s coated portion are incubated oven at 95 °C for next 5 hrs to allow hydrothermal growth of uniform nanorods. The prepared hydrothermally grown Nanorods were cleaned with isopropanol and deionized water to remove residual salts prior to annealing in a furnace under ambient air at 300 °C for 2 hrs for crystallizations. AuNP’s (purchased from Cytodiagnostics, 80–90 nm) were concentrated by centrifuging them at >8000 g and re-dispersed using molecular grade ethanol to increase the solutions wettability. Varying concentration of AuNP’s relative to the ZnO molar concentration used during sol-gel synthesis (2.5%, 5%, 7.5%, and 10%) was added/deposited on top of the ZnO-Nanorods. After 10 mins settling time, the Nanorods deposited with AuNP are treated at 60 °C for 20 mins to remove any residual ethanol solvents as well as to improve adhesion of AuNP onto the Nanorods surface.

### DNA capture probe immobilization and viral target hybridization

The earlier designed E6 forward primers are used as the capture probe of the ZnOAu-Nanorods IDE-based biosensor system. The thiolated probe is immobilized onto the ZnOAuNP-Nanorods by adding 10 µl of 1 µM of thiolated ssDNA probe on top the coated chip surface. The chip is then placed within a wet chamber, at RT for 3 hrs straight. After 3 hrs, the chip is then rinsed with PBS and TE-buffer solution through a micropipette manner of agitations followed by air drying to remove unbound probes. Chips with immobilized ssDNA probes are kept in 4° chiller. 10 µl of genomic DNA extracted from HPV-16 positive cancer cell lines (E.g. *CaSki*) are then added to chips surface and left for the hybridizations to take place at RT for 10 mins (inside wet chambers). PBS is used to wash the surface to remove any remaining, unbound gDNA strands followed by air drying. Binding or hybridization of viral DNA to the E6 capture probes are further analyzed and measured through electrochemical impedance spectroscopy (EIS).

### Detection of HPV-16 E6 viral DNA through EIS measurements

EIS measurements are done using Novocontrol alpha high-frequency analyzer (Hundsangen, Germany). Four individual EIS measurements were taken for bare IDE chips, ZnOAu-Nanorod coated chips, probe immobilized chips and target hybridized chips. Each measurement is taken by immersing the chip’s active sensing area in 0.1 M PBS buffer (pH 7.4) containing a mixture of 2 mM K_3_(Fe(CN)_6_)/K_4_(Fe(CN)_6_), placed on top the chips at RT. The impedance spectra of the real and imaginary parts of impedance, Zs’ and Zs” respectively were obtained and recorded by sweeping the frequency of 1–100 MHz with an applied AC amplitude of 1 V RMS. Nonlinear curve fitting is performed for the obtained EIS results using Randles equivalent circuit through ZView software. The obtained charge transfer resistance (R_ct_) values are subsequently recorded.

### Evaluating the analytical performance of the HPV-16 biosensor

Analytical performances such as signal response, sensitivity, specificity, stability, and reproductivity were done using the same steps/protocols mentioned above. The sensitivity of the fabricated HPV-16 biosensor is analyzed using a range of HPV-16 genomic DNA concentrations ranging from 10^−7^ to 10^−15^. The specificity of the biosensor was evaluated using single, triple base pair mismatch and non-complementary E6 genes. Stability of the modified chips was also analyzed using the same ZnOAuNP-Nanorods coated IDE chips by measuring the EIS outputs and the respective R_ct_ values once in a week for the next 4 weeks successively. Reproducibility of the detection signals was also analyzed by measuring the EIS signal outputs and R_ct_ values multiple times, at least 3–4 times during each analytical test using the same HPV-16 E6 viral DNA target.

## Data Availability

Authors declare that the materials, data, and protocols provided are promptly available to readers.
